# Progress in Gene-Editing Technology of Zebrafish

**DOI:** 10.3390/biom11091300

**Published:** 2021-09-01

**Authors:** Yanling Li, Zhipeng Jia, Shuchao Zhang, Xiaozhen He

**Affiliations:** Institute of Life Sciences, College of Biological Science and Engineering, Fuzhou University, Fuzhou 350108, China; 18768981502@163.com (Y.L.); jiazhipeng2021@163.com (Z.J.); m18336494258@163.com (S.Z.)

**Keywords:** zebrafish, *Danio rerio*, gene editing, double-stranded break, nick, genome modification, fixed-point orientation transformation, vertebrate model

## Abstract

As a vertebrate model, zebrafish (*Danio rerio*) plays a vital role in the field of life sciences. Recently, gene-editing technology has become increasingly innovative, significantly promoting scientific research on zebrafish. However, the implementation of these methods in a reasonable and accurate manner to achieve efficient gene-editing remains challenging. In this review, we systematically summarize the development and latest progress in zebrafish gene-editing technology. Specifically, we outline trends in double-strand break-free genome modification and the prospective applications of fixed-point orientation transformation of any base at any location through a multi-method approach.

## 1. Introduction

Zebrafish (*Danio rerio*) is genetically similar to humans [1] and has unique advantages in the field of vertebrate development, organ formation, gene function, and organ regeneration, mainly because of its small body size, transparent embryos, ex vivo fertilization and embryogenesis, rapid embryological development, low breeding cost, and high fecundity [2]. In addition, zebrafish is currently the only vertebrate suitable for microplate high-throughput drug screening, the application of which can be promoted if zebrafish are used to create different disease models. Numerous zebrafish models of human diseases can be constructed through drug immersion or physical injury. However, genetic zebrafish models of human diseases associated with gene mutations must be created through genetic modification. With the development of gene editing technology, an increasing number of gene-editing methods have been proposed and applied. Continuous exploration and improvement of gene-editing methods are important for the advancement of life science research and the promotion of gene therapy.

From the perspective of gene editing, the advantages of zebrafish over other species include high fecundity, ex vivo fertilization, easy injection, and convenient genotyping. The challenging task is that early-stage embryos can develop and split rapidly; therefore, gene editing has a relatively short working time, and an editing system of mRNA and protein components must be employed to improve efficiency. Various editing methods in zebrafish are under development; however, some challenges need to be overcome. A summary and review of the current gene-editing methods will help us understand and improve the use of the existing gene-editing methods in zebrafish. For better reading, the abbreviations involved in this review are organized in are organized in Abbreviation.

## 2. Transgenic Technology

DNA recombination technology started developing in the 1970s, marking a new era in biology. In the 1980s, with the successful application of transgenic technology in fish, a milestone in fish gene-editing technology was achieved [3]. Originally, the target gene, including plasmid DNA and bacterial artificial chromosomes, was introduced into the organism to meet human demands [4,5]. Traditionally, linearized exogenous DNA was injected into fertilized eggs alone to realize the transgene. However, the integration efficiency of foreign genes into the genome and the probability of transmitting the DNA to offspring is very low [5]. Moreover, the technique is non-directional, and the presence of multi-exogenous plasmid copies may lead to abnormal development of fertilized eggs, a large number of malformed offspring, and difficulty in integration site detection. Therefore, establishing transgenic lines in zebrafish was a difficult task.

More recently, transposase or macronuclease I-SceI-mediated transgenic methods have been developed, which can significantly improve the efficiency of transient and stable expression of zebrafish genes [6]. When a transposon, reporter gene, and promoter with a transposase are co-injected into the embryo, researchers can successfully insert the reporter gene into the genome [7,8].

DNA transposons are mobile genetic elements whose insertion positions can change within the host genome [9]. The transposon can be cut off from its original position and inserted into a new genomic position through catalysis by a transposase, forming a forward repeat at the target point. 

In addition to the advantages of high transgenic efficiency, transposons can also be used as sequence tags for inserted genes. Reverse PCR can facilitate the detection of insertion sites of exogenous genes, and Cre/LoxP technology can be used for efficient site-specific operations [10]. With the advancement in research, transposons have been widely employed for gene trapping. For example, Suster and colleagues have reported the use of the Tol2 transposon to capture genes in zebrafish [11]. The genomic sequences upstream and downstream of the insertion site of the trap vector can be identified by reverse PCR, and fusion transcripts of reporter genes and endogenous genes can be obtained by rapid amplification of cDNA ends (RACE) [12]. By using integrase (ex. phiC31), researchers can avoid the positional effects and multiple insertion of exogenous DNA into the genome [13].

The use of transposons has significantly improved the efficiency of transgenes in zebrafish, and a large amount of transgenic zebrafish strains have been developed accordingly. Tol2 and *Sleeping Beauty* transposons, found in fish, can form a genetically modified zebrafish strains [12]. For instance, some transgenic zebrafish lines show cell- or tissue-specific expression of commonly used fluorophores, such as green fluorescent proteins (GFP), which can provide valuable insights into gene function, organ formation, and cell behavior during development [14,15].

## 3. Targeting Induced Local Lesions in Genomes (TILLING)

The approaches in the above studies represent forward genetics. Given that zebrafish can also be used to establish a specific gene disease model to screen drugs for related diseases, reverse genetics approach would be more suitable for pre-clinical research. In recent years, the zebrafish has become a reliable model for reverse genetic analysis of vertebrate development and human diseases [16,17].

TILLING is a reverse genetics strategy that identifies mutations in specific genes of interest in chemically mutagenized populations [18]. The method was first described in *Arabidopsis thaliana* in 2000 [19] but was rapidly implemented in other organisms including zebrafish [20]. The approach consists of screening individual genomic DNA samples from a cohort of ENU-mutagenized F1 zebrafish to identify mutations that alter a chosen gene, while the sperm of the corresponding fish is cryopreserved for subsequent reconstitution of the mutant line by in vitro fertilization once desired mutations are identified.

TILLING is a powerful technology that raises zebrafish as a pertinent model in gene function research. However, the procedure of TILLING is costly, labor intensive, and cannot be implemented in most individual labs. The most important thing is that the identification of mutant genes is very troublesome, and whether there are multiple mutation sites cannot be well determined [21]. 

## 4. Discovery and Application of Fixed-Point Shear Enzymes

In 1983, the first zinc finger protein (ZFP) domain was identified in the transcription factor IIIA (TFIIIA) from *Xenopus laevis* [22]. In 2008, a zinc finger nuclease (ZFN) was designed to recognize the homologous sequence of *vascular endothelial growth factor receptor 2* (*kdrl*) in zebrafish [23]. The technique using ZFN relies on the specific recognition and binding of DNA ZFP, and the cleavage domain of FokI endonuclease enables reverse genetics in zebrafish [23,24,25,26,27]. Gene mutation was successfully induced by injecting ZFN mRNA into the one-cell stage zebrafish embryos to generate double-strand breaks (DSBs) at the target site. However, the design of ZFN is difficult, expensive, and inefficient for certain targets [28,29,30], which hinders the development of this technology in zebrafish. Designing and screening specific ZFNs requires time and numerous experiments. If the specific binding site of DNA is invalid or FokI homodimerization cleavage is difficult, an off-target effect may occur, thus limiting the large-scale application of this process [24,31]. 

The transcription activator-like effector nuclease (TALEN) is another type of genome targeting nuclease after ZFN, which is more flexible and efficient [32,33]. In 2007, a novel DNA-binding protein called the transcription activator-like effector (TALE) was identified in a Gram-negative plant pathogen. TALE is a class of protein effectors that can be injected into host cells by *Flavobacterium* through the secretion system [34]. TALEN is constructed using the same method of constructing ZFN and can target and modify genomes conveniently and efficiently. ZFN and TALEN are artificial nucleases composed of specific DNA-binding proteins and non-restricted nuclease FokI [35,36]. Their mechanism of action is similar: FokI exhibits endonuclease activity by forming dimers, causing DSBs in the target DNA sequence and subsequently inducing endogenous repair mechanisms of cells. This activates non-homologous end joining (NHEJ) or homologous recombination (HR) in vivo that leads to endogenous gene knockout or exogenous fragment knock-in (KI) at the target site [37]. In 2011, RNA encoding different TALEN pairs was injected into one-cell stage zebrafish embryos for the first time [31]. All four pairs of TALENs induced targeted indels with high mutation frequency ranging from 11% to 33%. These mutations were caused by TALEN-induced DSB repair through NHEJ, which resulted in effective indels at the breaking site. These indels can cause frameshift or knockout mutations that can be passed on to the next generation [31]. ZFN and TALEN can thus mediate targeted genomic modifications in vivo, enabling the development of genetic studies and disease models. The emergence of targeted ZFN and TALEN technology has improved the efficiency and success rate of targeted gene modification as well as recognition specificity [38]. 

The gene tool clustered regularly interspaced short palindromic repeats (CRISPR) and CRISPR-associated protein (CRISPR/Cas) is economical, convenient, and efficient [39,40,41] and requires only one guide RNA (gRNA) to be customized for a specific sequence [42] rather than two ZFN or TALEN proteins that must be designed and assembled for each site. gRNA is approximately 100 bp, and it is thus easier to construct compared with that of ZFN or TALEN. Moreover, because of its short length, complications caused by the long encoding vector can be avoided. The CRISPR/Cas repeating structure was first discovered in 1987 in the flanks of the *iap* gene sequence from *Escherichia coli* [43] and was named short regularly spaced repeats in 2000 [44]. By 2013, an expression vector to produce Cas9 mRNA by a SP6 RNA polymerase and a customizable single guide RNA (sgRNA) that consists of a 20 bp nucleotide sequences complementary to the target site was used to construct the CRISPR/Cas9 genome editing technology in zebrafish [32]. The Cas9 capped mRNA and sgRNA were co-injected into one-cell stage embryos to effectively introduce somatic indel mutations at 8 out of 10 sites in the zebrafish genome, and the average mutation frequency of these eight loci ranged from 24.4% to 59.4% (Table 1) [32]. Therefore, CRISPR ushered in a new chapter for zebrafish gene-editing technology. Nevertheless, CRISPR/Cas9 also faces a major challenge regarding off-target effects that result from the tolerance of several base mismatches between the targeted DNA and the 20 bp sgRNA [45]. For clinical application, complete accuracy is required; therefore, it is particularly important to improve the specificity of sgRNA and change the targeted cutting mode [46,47,48]. In addition, donor type and targeted editing efficiency are important factors that limit zebrafish gene editing [49].

## 5. Knockout Gene Editing

Zebrafish is a model animal capable of rapid verification of candidate disease genes. Gene knockout is an important means of gene function verification. Fortunately, ZFN, TALEN, and CRISPR/Cas9 gene editing techniques can all knockout target genes in zebrafish. ZFN and TALEN, which can induce targeted mutations, can achieve certain effects in zebrafish as the DSB generated is subjected to error-prone repair or inaccurate repair such as via NHEJ [50]. In 2008, ZFN was designed to target the *kdrl* gene with a somatic mutation frequency of 20%, and the mutation can be transmitted to the offspring (Table 1) [23]. In the same year, disrupted *ntl* alleles were transmitted from ZFN-targeted founder fish in over half the adults tested at frequencies averaging 20% (Table 1) [25]. Using ZFN (Table 1) made by Oligomerized Pool Engineering (OPEN), five endogenous zebrafish genes, *tfr2*, *dopamine transporter*, *telomerase*, *hif1aa*, and *gridlock*, were successfully engineered [26,51]. In 2011, modularly assembled ZFN was applied to zebrafish and generated new germline mutations in eight different genes (Table 1) [24]. An optimized two-finger archive for ZFN was also used to introduce lesions at 9 of 11 target sites in the zebrafish genome (Table 1) [27]. 

In 2011, Sander and colleagues applied an engineered TALEN for gene targeting in zebrafish and demonstrated high efficiency that approached 33% at some loci (Table 1) [31]. In 2012, TALEN was also employed to edit zebrafish genes with an average somatic mutation frequency of 29.5% (Table 1) [52]. Large mutations can be induced by deleting ATG starting sites or promoting front-end frameshift mutations to produce termination codons within 50 amino acids at the target sites. However, numerous targeted indel mutations can be recovered in adult fish. 

Since then, CRISPR/Cas9 was introduced as a new generation of site-specific gene-editing technology. Studies have shown that CRISPR/Cas9 technology is one of the most effective gene-editing methods for zebrafish, regardless of cost, design, or reproductive heritability [53,54,55]. This method can also effectively delete large fragments and edit multiple sites simultaneously. In 2013, an improved CRISPR/Cas system was introduced for zebrafish to effectively target the reporter gene in transgenic line *Tg (-5.1 mnx1: EGFP)* and four endogenous loci, with a mutation rate of 75–99% [56]. The gene mutation induced by Cas9 can be transmitted to F1 offspring. Five genomic loci can be efficiently destroyed simultaneously, and the F0 generation shows multiple biallelic knockout phenotypes. Thus, this system is an efficient gene knockout method for zebrafish, and its high mutation rate may be attributed to the optimized zebrafish codons and double nuclear localization signals (NLS) tags of Cas9, which enable better expression and localization of the Cas9 protein [56]. In 2020, a simple method for massive deletion mutations of zebrafish gene lines was created using the CRISPR/Cas9 system, successfully achieving a deletion of up to 78 kb and simultaneously mutating two genes in one injection (Table 1) [57]. This technique can be employed when a single gRNA has no obvious phenotype or indel mutation. Furthermore, using the U6 promoter to transcribe gRNA and a tissue-specific promoter to limit the expression of Cas9, the CRISPR/Cas9 system can also be used to generate tissue-specific knockout [58]. Under the control of the Gal4/UAS system, tissue-specific expression of Cas9 and gene disruption can also be achieved [59].

In 2018, a system for gene knockout that consistently produces null phenotypes in G0 zebrafish was described [17]. Yolk injection of sets of four CRISPR/Cas9 ribonucleoprotein (RNP) complexes redundantly targeting a single gene recapitulated germline-transmitted knockout phenotypes in >90% of G0 embryos for each of eight test genes. Moreover, the durable effects of four guide Cas9 RNPs targeting *tyr* is better than single-guide Cas9 RNP. Simultaneous dual-gene knockouts can be generated with acceptable toxicity using combined guide sets in at least some cases. This system provides a platform for rapid screening of genes of interest in development, physiology, and disease models in zebrafish.

ErCas12a, a new member of the Cas12a family, was shown to successfully induce indels in zebrafish embryos (Table 1) [60]. ErCas12a mRNA was co-injected with noto-pre-crRNA1 or noto-pre-crRNA3 into one-cell stage zebrafish embryos, followed by heat shock at 34 °C for 4 h. The mutation efficiency of the *noto* targets was analyzed using Illumina next-generation sequencing (NGS), and 61% of the alleles of the noto-pre-crRNA1 target sites showed indels, whereas 90% of the alleles of the noto-pre-crRNA3 target sites showed indels, resulting in the phenotypic characteristics of *noto* biallelic deletion.

In 2019, all genes (1333) on chromosome 1 of zebrafish were systematically knocked out using CRISPR/Cas9 technology, resulting in a mutation of 1029 genes, and generating 1039 lines of genetic alleles corresponding to 636 genes (Table 1) [61]. Bioinformatic analysis showed that the success rate of gRNA targeting specific genes was positively correlated with the GC content of the target sites. Nearly a quarter of mutations are linked to human diseases. After introducing gRNA and Cas9 mRNA into zebrafish embryos, 962 coding genes and 67 non-coding genes were detected, 77.2% of which were successfully mutated. This study is a classic example of using CRISPR/Cas9 technology to perform reverse genetics in zebrafish, demonstrating the broad application prospects of CRISPR as an emerging technology.

In 2021, Isiaku and his colleagues [62] coupled well-characterized neutrophil- and macrophage-specific Gal4 driver lines with UAS:Cas9 transgenes for selective expression of Cas9 in either neutrophils or macrophages (Table 1). They injected two gRNAs targeting *trim33* into *Tg (mpx-Cas9)* and *Tg (mpeg1-Cas9)* lines. Sequencing results showed highly effective on-target gene editing in neutrophils but not in other cells from the same embryos. The NGS results of purified neutrophils from these embryos demonstrated a maximal nucleotide deletion incidence of 43.6% at the PAM cut site, and all resulted in transcripts predicted to encode Trim33 protein with early carboxyl truncations.

Gene targeted knockout is an efficient reverse genetics tool that has greatly enhanced our ability to analyze gene function in zebrafish. However, about 80% of zebrafish engineered mutants do not display a discernible phenotype [63]. This relative normal development in many mutants is attributed to the genetic compensation response (GCR). Thus, to unmask the phenotype in these mutants is very important to study the mutated gene function. Recently, two studies in zebrafish provide some solutions for gene mutation design, which are as follows [63,64,65]: (1) Generating mutants with a deletion of either full-locus or promoter; (2) generating mutants with an in-frame deletion in a critical domain of the protein; (3) generating mutants with a nonfunctional Premature Termination Codon (PTC) mutation in the last exon containing a critical domain; (4) crossing the mutant with a GCR with *upf3a^−/−^* mutant to construct a double mutant, as an *upf3a^−/−^* mutant develops relatively normal and remains fertile.

**Table 1:** Summary of gene knockout (KO) studies using ZFN, TALEN, and CRISPR/Cas9 in zebrafish.

Targeting System	Somatic KO Efficiency	Germline Transmission Rate	Reported in References
ZFN	10–20%	~30% (6/20)	[23]
ZFN	0.5–2%	1.3–25%	[24]
ZFN	2–32%	~20%	[25]
ZFN	0.4–15.7%	ND	[27]
TALEN	11–33%	ND	[31]
CRISPR/Cas9	2.7–72%	ND	[32]
ZFN	3–20%	6–50%	[51]
TALEN	20–77%	ND	[52]
CRISPR/Cas9	1–27%	22–33%	[57]
CRISPR/ErCas12a	24–90%	ND	[60]
CRISPR/Cas9	2–100%	ND	[61]
CRISPR/Cas9	27–84%	ND	[62]

ND, not determined.

## 6. Fixed Point-Oriented Reconstruction

**Figure 1:**
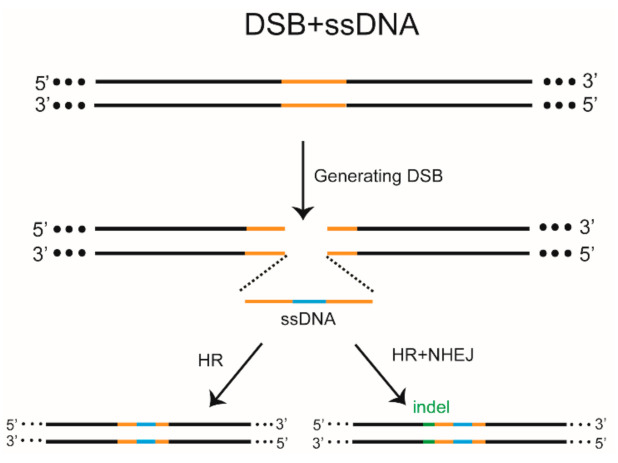
Double-strand breaks (DSB) and single-stranded DNA (ssDNA) mediated gene editing in zebrafish. ZFN, TALEN, or CRISPR/Cas9 can be used to generate DSBs at the target site. ssDNA serves as a template for repair. Homologous recombination (HR) precisely adds exogenous fragment at the cut site. Alternatively, the 5′ end undergoes error-prone non-homologous end joining (NHEJ), leading to indel production.

There are at least two DSB repair mechanisms induced by ZFN, TALEN, or CRISPR/Cas9 systems: (1) HR and (2) NHEJ [66]. HR is an accurate repair method, in which the long homologous arm sequence in the donor serves as a template for complementary recombination with the target gene sequence [67,68]. NHEJ-mediated repair is the direct adhesion after a gap in the DSB, generating a different indel at the targeted site (insertion or deletion) and disrupting the gene [69]. The NHEJ pathway is also divided into classical NHEJ (c-NHEJ) and alternative NHEJ (alt-NHEJ), also known as microhomology-mediated end joining (MMEJ) [70]. c-NHEJ is active in all phases of the cell cycle, whereas MMEJ is similar to HR and is more active in the S and G2 phases. In the repair mechanism, a 3–30 bp microhomologous sequence is annealed at the DSB site, and the redundant DNA is deleted, resulting in a small deletion mutation at the targeted site. The efficiency of HR-mediated reporter gene insertion can be improved with a homologous arm, and the simultaneous breakage of the targeted sites and donor under the action of Cas9 can enhance the efficiency of reporter gene integration [71]. Studies have shown that KI mediated by CRISPR/Cas in zebrafish can be achieved by homology-independent DNA repair [72,73,74]. Researchers have also integrated foreign genes into the genome through MMEJ [75]. In summary, targeted transformation of the zebrafish genome can be achieved using a donor. Therefore, the structure, purity of the donor, and the length of the homologous arm can affect the efficiency of KI [76,77,78]. Single-stranded oligonucleotide (ssODN) donors tend to produce numerous non-target mutations at the cleavage sites (Figure 1), and thus double-stranded DNA (dsDNA) is generally chosen as the donor template [79]. dsDNA templates with complete nuclease cleavage sites can improve the integration efficiency, but they increase the risk of random insertion [80]. Therefore, circular plasmids appear to be the better donor template [55]. Some researchers have attempted to optimize the length of the donor homologous arm and found that a long homologous arm of ~2 kb is efficient for HR-mediated KI [68,79]. At present, most gene editing in zebrafish is initiated by DSBs.

## 7. DSB-Mediated Gene KI through NHEJ

NHEJ is an error-prone process that can effectively introduce indels, which result in frameshift mutations or premature termination of the coding region, disrupting protein coding and thereby damaging critical regions in the genome.

In the early developmental stage of zebrafish, NHEJ is the main repair mode for DSB and is at least 10 times more active than is HR [81]. c-NHEJ generally handles numerous repairs, which depends on the ku70/80 heterodimeric, DNA-PKCS, XRCC4/LIG4, and cofactors, including XLF, WRN, Artemis, APLF, PNKP, and APTX [82]. When c-NHEJ is not activated, DSB repair is performed by alt-NHEJ, and the participating factors are PARP1, LIG1, LIG3, and PoI Theta [83,84]. When NHEJ occurs, the two broken parts will be connected directly or some bases will be inserted/deleted randomly, which may cause a frameshift mutation [85].

In 2014, researchers reported that long DNA fragments can be integrated into the zebrafish genome through NHEJ using the CRISPR/Cas9 system [73]. The donor plasmids, sgRNAs (one for genome splicing, the other for donor plasmid splicing), and Cas9 mRNA were co-injected into the embryos to cut the genomic DNA and donor plasmid simultaneously, resulting in the incorporation of the donor plasmid into the genome. EGFP was then successfully transferred to the Gal4 strain fish, increasing the possibility of using CRISPR/Cas9 to produce transgenic zebrafish efficiently [73]. However, no stable KI transgenic fish incorporating exogenous genes into endogenous genomic loci was obtained. In the same year, Kimura et al. [74] improved the aforementioned method and successfully produced KI transgenic zebrafish with a reporter gene or with Gal4 expression (Table 2). The efficiency of producing KI-fish at the *evx2* and *eng1b* loci was 12% (2/17) and 3% (1/40), respectively. When effective sgRNAs are identified, embryos extensively expressing fluorescent proteins can be cultured to produce transgenic lines with an efficiency of over 25%. However, because of the insertion of exons or *cis*-regulatory elements of the target genes, the coding sequence of the targeted endogenous genes is disrupted, and the expression of inserted exogenous genes is not desired.

In 2015, Li et al. [86] used the CRISPR/Cas9 system to develop an efficient KI method targeting zebrafish introns, independent of HR (Table 2); this method can maintain the integrity of the coding sequence and the regulatory elements of endogenous target genes. The team designed an sgRNA targeting the last intron of *tyrosine hydroxylase* (*th*) in zebrafish and co-injected Cas9 mRNA and sgRNA into the one-cell stage zebrafish embryos, with a cutting efficiency of approximately 83%. Subsequently, a donor plasmid, th-P2A-EGFP, was designed, which does not disrupt Th expression. Both the donor plasmid DNA and the last intron of *th* contain sgRNA targets. Therefore, the simultaneous cleavage of both enables exogenous DNA to be specifically integrated into the *th* site in a non-HR manner. When the F0 generation is hybridized with wildtype zebrafish to screen the F1 generation, the production rate of EGFP-positive F1 offspring ranged from 15.5% to 21.1%. 

In 2017, four fluorescent reporter gene KI lines at *otx2* and *pax2a*—two key genes involved in midbrain–hindbrain boundary development—were generated using CRISPR/Cas9 (Table 2) [87]. The coding sequence of the fluorescent protein Venus or tRFP was knocked upstream of the corresponding ATG, thus its expression was driven by the endogenous promoter/enhancer. Moreover, the exogenous fragment did not interfere with the expression of the endogenous gene. The germline transmission rates were 20% for Pax2a:venus and 2.8% for Pax2a:tRFP reporters.

In 2018, CRISPR/Cas9 technology was applied to integrate mCherry into the final coding sequence of the target gene with a 25 bp microhomologous arm to efficiently generate seamless zebrafish KI lines (Table 2) [88]. This seamless KI technology maintains the integrity of endogenous genes and does not damage the function of the target gene. At the *fli1a* and *gata1a* loci, the precise integration efficiency mediated by MMEJ was 22% and 24% as observed by F1 fluorescence screening, respectively.

In 2020, GeneWeld, a homology-mediated end joining (HMEJ) strategy for targeted integration directed by short homology at high frequency at a DSB site, had high rates of germline transmission (22–100%) for targeted KIs at eight zebrafish loci. The reason for high efficiency benefits from plasmids for Gene Tagging (pGTag) containing reporters flanked by a universal CRISPR sgRNA sequence, which enables in vivo liberation of the homology arms [98].

**Figure 2:**
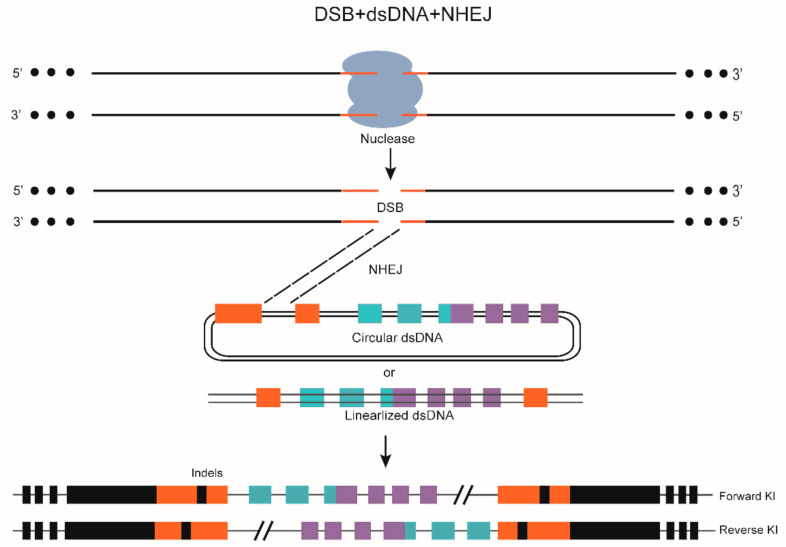
DSB and dsDNA mediated gene editing in zebrafish. ZFN, TALEN, or CRISPR/Cas9 can be used to generate DSBs at the target site. Linearized or circular dsDNA containing the DNA fragment serves as the template. Targeted knock-in (KI) can then proceed via the NHEJ pathway. In addition to forward KI, reverse KI and indels often occur.

Long fragment gene KI through NHEJ has certain disadvantages, such as random integration risk, imprecise insertion position, and non-in-frame insertion mode (Figure 2). For instance, in a 2014 study, approximately 3% (9/388) of RFP-expressing cells in RFP-positive embryos were located outside the neurod:EGFP-expressing region (in muscle or skin cells) after targeting *Tg (neurod:EGFP)* × *Tg (UAS:RFP, cry1:EGFP)* embryos [73]. In addition, the off-target integration of donor plasmid events (about 0.3%) was further confirmed when targeting the *Tg (UAS:RFP, cry1:EGFP)* embryos with neurod:EGFP. In the same year, a reverse insertion was verified when targeting the *evx2* site in the *Tg (UAS:RFP)* embryos [74]. Although the efficiency of NHEJ-mediated KI is generally high, unwanted indels always occur, hindering the development of this method.

## 8. DSB-HR-Mediated Gene KI

In contrast to the simplicity, uncontrollability, and randomness of NHEJ-mediated KI, HR-triggered KI is a much more complex and accurate tool for fixing and editing the genetic sequence of the targeted organism. HR is mainly active in the S and G2 phases of the cell cycle and competes with NHEJ when repairing DSB [99]. At the initial DSB repair stage, the excision of DNA ends required for HR initiation determines pathway selection when both HR and NHEJ are activated [100]. In mammalian cells, the key NHEJ factor, Ku, is one of the first factors that binds the broken ends of DNA [101]. Subsequently, the RIF1/53BP1 complex can maintain Ku binding and block resection, promoting NHEJ, or the CtIP/BRCA1 complex [102] can stimulate resection with PCNA, EXO1, BLM, and MRE11/RAD/NBS1 (MRN) complexes, leading to the removal of Ku from the DNA end and promoting HR [103].

In addition to the functional deletion of genes, ZFN, TALEN, and the CRISPR system can be used to perform more subtle genomic modifications in zebrafish via HR or NHEJ-mediated KI. In 2012, single-stranded DNA (ssDNA) was first introduced to promote HR-mediated KI in zebrafish (Table 2) [89]. Targeted sites in the zebrafish genome were accurately modified using the improved Goldy TALEN system under recombination action using ssODN. The mutation rates of the *ponzr1* site were increased by almost six-fold, and the germline heritability increased from 17% to 71%. EcoRV and modified loxP (mloxP) fragments were introduced into somatic tissues, and the genetic frequency of mloxP was 10.3% on *crhr2*. ssODN donors can also co-create precise targeting sequence correction with RNA-guided nucleases using the CRISPR/Cas system [104]. At the *fh* and *gsk3b* targeting sites, sense ssDNA was used as a donor to insert 3–4 bp fragments, with accurate modification rates of 8.3% and 1.7%, respectively. In 2016, the ssODN donor template for site-directed single-nucleotide editing was introduced to target *tardbp* and *fus*, two disease-related genes, with the CRISPR/Cas9 system. Subsequently, 46 adult zebrafish were hybridized with wild-type zebrafish and only one was found to inherit the correct editing sequence of the Tardbp A379T mutation (Table 2) [90]. This method proved feasible for site-directed single-nucleotides editing in zebrafish [105]. In 2020, the zebrafish long single-stranded DNA template (zLOST) was introduced to produce more efficient and accurate mutations in zebrafish by utilizing HR with a long ssDNA template (Table 2) [91]. Using zLOST containing *tyrosinase* (*tyr*) repair sites, nearly 98% of Albino tyr25del/25del embryos recovered their pigmentation. The heritability of this method is 31.8%. Although precise repair events can be detected in most targeted alleles using DSB and ssDNA, many targeted alleles undergo inaccurate and error-prone repair steps, which may involve the NHEJ repair mechanism. Moreover, the length of the integration sequence in this method is limited due to the instability of ssDNA [89].

In 2013, the “unit assembly” method was introduced to rapidly construct transcriptional activator-like effector repeats, and a linearized dsDNA donor with long homologous arms was employed to accurately modify three endogenous loci (*th*, *fam46c*, and *smad5*) in zebrafish (Table 2) [38]. However, the germline transmission rates of HR events at the *th* site were approximately 1.5%; the lower germline transmission efficiency may be due to the application of linearized dsDNA, which harbors inherent toxicity. Nevertheless, this approach can precisely introduce modifications to any desired site in the zebrafish genome, significantly expanding the utility of this model. 

Circular dsDNA with a sgRNA-targeted recognition sequence at both ends of the homologous arm to linearize the donor in vivo was also applied to improve the HR efficiency (Table 2) [79]. Almost 50% of the larvae with the exact homologous repair of the *alb* gene were obtained using circular donor DNA and approximately 10% fish passed the repaired allele onto the next generation (3/28 adult fish). Donor DNA containing long homologous arms also proved to be efficient (2/25) in inserting the fluorescent reporter gene or KalTA4 transactivator into the endogenous *nefma* gene via DSBs, followed by HR; this made it more convenient to study the nervous system with CRISPR/Cas technology (Table 2) [92].

Short homologous arms have also been employed; for example, in 2019, the CRISPR-ErCas12a system was used to integrate a reporter gene into the zebrafish genome using short homologous arms and achieved an average of 31% embryos with the GFP signal [60].

The disadvantages of HR-mediated KI of exogenous genes generated by DSB repair include the generation of unwanted indels, multiple copy risk, short-fragment insertion, and low germline genetic efficiency [39,104,106].

## 9. Inevitable Side Effects of DSBs

DNA damage caused by DSB triggers P53 induced apoptosis, affects the stability of the genome, increases carcinogenesis risk in the edited cells, and occasionally generates a genetic compensation mechanism [107]. In the absence of a donor, DSBs are mainly repaired by NHEJ, which is generally error-prone and accompanied by indels. On-target and off-target mutations caused by DSBs can lead to allelic and non-allelic mutations, loss of heterozygosity, translocation, and other unwarranted genetic changes [108]. Moreover, KI via the DSB repair pathway is not very efficient, and the off-target phenomena remain a challenge. The DNA repair pathway can be regulated by injecting small molecules (NU7441) directly into fertilized zebrafish embryos to inhibit NHEJ and increase CRISPR-mediated HR by 13.4-fold; however, the wide applicability and possible side effects of this method need to be further analyzed [109]. In our opinion, generating DSBs is not an ideal method for achieving seamless gene editing as it may jeopardize phenotypic experiments, genetic procedures, and even clinical applications. Therefore, alternative approaches should be explored. To avoid off-target effects, modified Cas9 nickases (Cas9n) or dead Cas9 (dCas9) can be used for the initial stage of gene editing. Moreover, editing without DNA components is becoming increasingly popular.

## 10. Nickase System

**Figure 3:**
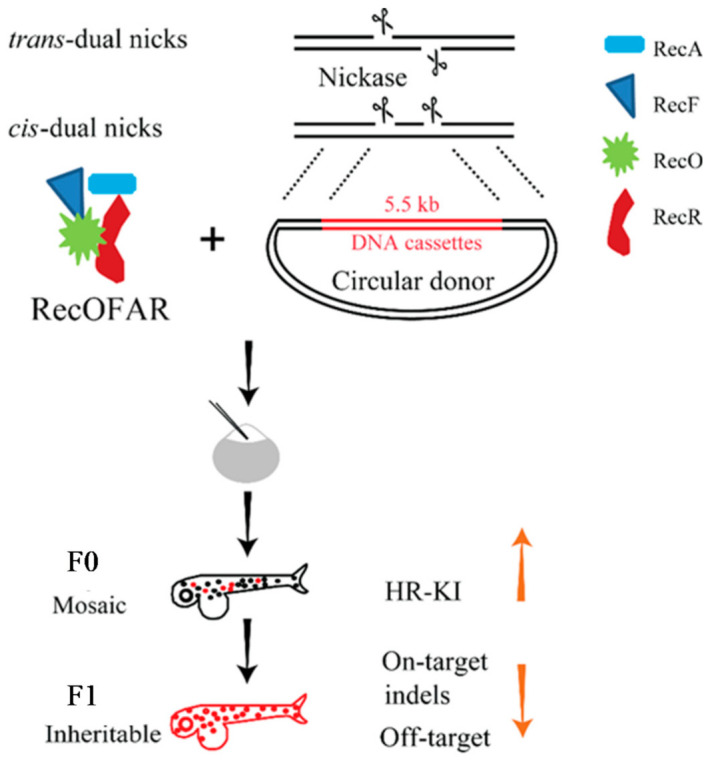
Nickase-based homologous recombination enhanced by recOfar factors (NEO) mediated gene editing in zebrafish. The genome editing strategy is called nickase-based homologous recombination enhanced by recofar factors (NEO). The bacterial RecA protein together with RecO, RecR, and RecF factors can enhance accurate HR-KI induced by *trans*-dual nicks (two cooperative nicks induced on the complementary strands) or *cis*-dual nicks (both nicks on a same strand) in zebrafish. Furthermore, the NEO system can enable KI of >5.5-kb-long DNA cassettes into the zebrafish genome. In addition, both on-target and off-target indels that are prevalent when conventional Cas9 strategies are adopted could be substantially reduced via NEO in zebrafish. The upward yellow arrow indicates an increase in HR-KI efficiency. The downward yellow arrow indicates a decrease in the on-target and off-target indel count ratio.

Although HR is mainly related to DSB repair, some studies have shown that nicks can also initiate HR [110,111]. In previous studies published by our research group, DNA nicks were used as an alternative to DSB-initiated gene editing (Table 2) [93]. The D10A Cas9n system combining two sgRNAs can be used to generate dual nicks. For the first time, our team implemented a nick-based KI method to reduce off-target rates and optimize nick-mediated KI in zebrafish (Figure 3). A pair of sgRNAs was placed close to the stop codon of the *gfap* gene to produce *trans*- or *cis*-dual nicks, and the p2A-ChR2-EYFP reporter was fused. The integration efficiency of p2A-ChR2-EYFP was then determined by PCR and sequencing; the germline transmission efficiency of the *trans*-dual nicks was very low (2.7%), and that of the *cis*-dual nicks was 0%. Subsequently, the authors explored the recombinants, RecA and RecORF, from the bacterial system and found that RecOFAR can inhibit NHEJ events and promote HR repair. When RecOFAR mRNA, CRISPR/Cas9n, sgRNAs, and a circular donor plasmid (with a modified PAM site) were co-injected into the embryos, the germline transmission efficiencies of the *trans*-dual nicks and *cis*-dual nicks were increased to 11.1% and 9.4%, respectively. This method used dual nicks for the first time to achieve efficient genome site-specific long DNA fragments (>5.5 kb in length) KI in zebrafish. 

Establishing fish strains via endogenous genes modification is important. The KI method of generating nicks for HR repair can not only achieve insertion of long fragments, but it can also significantly improve accuracy while ensuring efficiency. Interestingly, *cis*-dual nicks do not appear to induce DSBs, suggesting that the HR repair pathway of nicks is different from that of DSBs. Nevertheless, this method relies on donor DNA with two long homologous arms (800–1000 bp), and the construction of the donor is time-consuming and costly. Therefore, short-fragment gene editing must be supplemented by other methods. At present, the trend is to adopt a point mutation editing technique that does not produce DSBs nor rely on a DNA template.

## 11. DNA Template-Free Techniques (Single Base Substitution)

The HR pathway can only achieve fixed-point replacement of bases when provided exogenous donor DNA. Currently, donor DNA is available three forms: Circular dsDNA, linearized dsDNA, and ssDNA [76,77,78]. However, no clear and unified method exists for the selection of donor DNA and the length of the homologous arm to achieve the best HR effect. Although the HR pathway can change any base, it is limited by cell type and cell cycle, and the efficient delivery of donor DNA into cells is also a significant issue. These disadvantages limit the use and application scope of HR in animals and plants. In addition, the NHEJ pathway competes with the HR pathway, which often leads to the unnecessary editing of products at the target [99]. Therefore, achieving efficient and stable single-base mutations is challenging in DSB-mediated HR. Moreover, HR generally occurs in dividing cells during the S and G2 phases of cells, and thus it occurs less frequently in plant and animal cells. Therefore, DNA-free template technology is a better choice.

**Figure 4:**
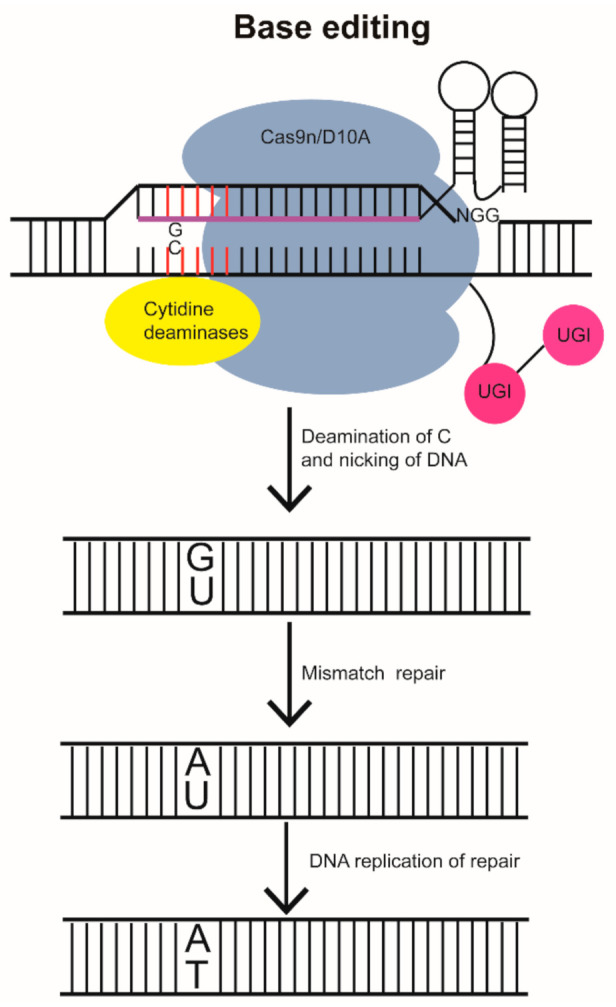
Base editing (BE) technology. Cytosine base editors consist of a deaminase and uracil glycosylase inhibitor (UGI) fused with the N and C terminus of D10A Cas9n, respectively. Deaminase can convert cytosine into uracil to produce a U:G wobble. UGI prevents the conversion of U back to C. U:A is formed during mismatch repair and is then converted to T:A through replication, thus completing the C:G to T:A substitution.

Currently, the base editing system—relying on a base editor (BE) that is a fusion protein of *Streptococcus pyogenes* Cas9 (SpCas9) and APOBEC1 in rats [112]—can be divided into cytosine base editors (CBEs) and adenine base editors (ABEs) depending on the different base-modified enzymes that are fused. These two base editors can achieve cytosine (C) or adenine (A) deamination reactions at a certain range of target sites using cytosine deaminase (Figure 4) or modified adenine deaminase, respectively, without DSB production and can achieve accurate C–T or A–G replacement by DNA repair or replication. Moreover, the base editing system does not depend on the generation of DSBs because the dCas9 protein with no cleavage activity or Cas9n with only one-strand cleavage activity is used to achieve site-specific base substitution at the target site [113,114]. However, although CBE can be used to edit the required C or G nucleotides into T or A, its overall efficiency is lower in various cells and organisms, including zebrafish [115,116]. This can cause unexpected nucleotide changes and indels at the target sites. 

In 2017, the cytidine deaminase-Cas9n fusion protein was used to achieve site-specific single-base mutations of up to 28% in multiple gene sites (Table 2) [94], but indels were produced in the zebrafish. In 2018, to reduce the formation of indels, dCas9 was used to replace Cas9n in the BE system, and the zABE7.10-dCas9 system was constructed but with a reduced A→G base conversion rate [95]. Therefore, Cas9n functions better than dCas9. Since then, BE3 has been significantly improved by developing an optimized set of CBEs known as BE, BE4max, and AncBE4max [95]. Somatic editing was observed in 4–11% embryos injected with BE4max and AncBE4max targeting *twist2,* and BE4max correctly edited one of the two targeted nucleotides in 9% and 3% of the embryos produced by two F0. However, most F2 progenies inherited the wrongly edited allele, and off-target editing was observed in embryos that inherited the correctly edited allele. Compared with BE4max, AncBE4max accurately edited the two nucleotides of *twist2*, with a 19% efficiency in F1 embryos. Although this method may cause off-target and incorrect editing, both BE4max and AncBE4max can effectively edit zebrafish strains without introducing indels [96]. The new ABE, ABEmax, with an improved NLS and optimized codons, was also applied to zebrafish and considerable efficiency was achieved (Table 2) [95,96].

Compared with HR, the base editing system can only recognize transition but not transversion and has a higher off-target effect on the genome-wide and RNA level [117,118]. There are two main reasons for the occurrence of such off-target effects. First, the fault tolerance of the sgRNA sequence and the off-target effects are related to the number of mismatched bases between the sgRNA and target DNA. Second, the non-specificity of the Cas9 nuclease, the high concentration of the deaminase-Cas9 fusion protein, and the long-term continuous expression of fusion proteins can easily lead to editing of off-target sites. Although base editing has shown reliable application potential for gene therapy of specific genetic diseases, it does not seem suitable for large-scale construction of zebrafish genetic disease models.

## 12. Prime Editing Technology

**Figure 5:**
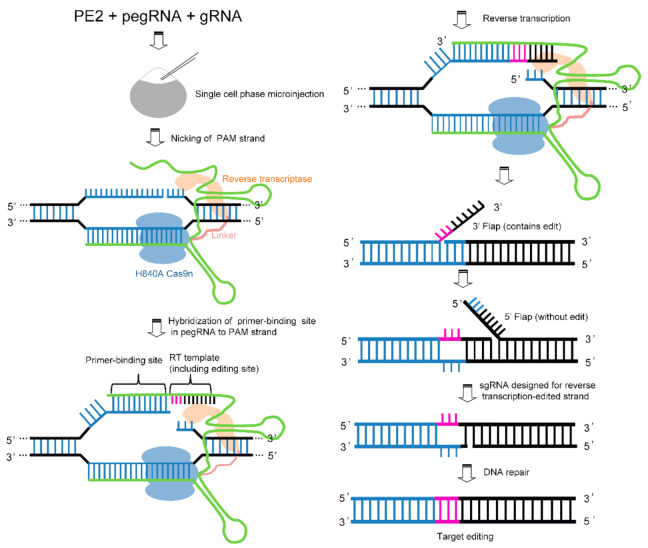
Prime editing technology. The prime editor (PE) is a H840A Cas9n and reverse transcriptase (RT) fusion protein coupled with a prime editing guide RNA (pegRNA). The PE2:pegRNA complex binds to the target DNA and nicks the PAM-containing strand. To improve editing efficiency, additional gRNA nicking of the complementary strand is preferred. During the repair process, the primer-binding site in pegRNA hybridizes to the PAM strand. The RT template of the pegRNA is converted into new DNA by RT. Under the action of proteins, equilibration between the edited 3′ flap and the unedited 5′ flap can be achieved. Finally, DNA repair results in stably edited DNA.

In many cell types and organisms, including mammals, base editing can achieve various transition mutations (C→T, G→A, A→G, and T→C) without producing DSBs. However, it cannot complete eight types of transversion mutations (C→A, C→G, G→C, G→T A→T, T→A, T→G, and A→C). Currently, the efficiency of HR in clinically related cell types is low, and numerous indels are observed. Compared with HR and base editing, prime editing has the advantages of high efficiency, fewer by-products, low off-target rates, and the ability to achieve targeted indels and 12 base conversions [119]. Prime editing includes two components, the prime editor (PE) and prime editing guide RNA (pegRNA). PE is a complex of Cas9n and M-MLV retroviruses while the pegRNA, based on sgRNA, extends the corresponding sequence at the 3′ or 5′ end to target the sequence to be edited and provides a reverse transcription template. The specific mechanism of sgRNA and Cas9n relies on generating a nick around the sequence to be edited, which triggers the formation of 3′ flap-5′ equilibration. In the subsequent repair process, pegRNA with template information is introduced, after which the participation of reverse transcriptase and endogenous Fen1 protein leads to the targeted transformation of the regional genome. Prime editing has significantly expanded the scope of genome editing (Figure 5). In principle, it can correct up to 89% of the known genetic variations related to human diseases [119]. Moreover, this method can efficiently establish many genetic disease models in zebrafish, significantly promoting the application of zebrafish in the study of human diseases. Recently, purified ribonucleoprotein complexes were introduced to construct somatic mutations in zebrafish embryos with frequencies as high as 30% and demonstrated germline transmission (Table 2) [97]. One challenge is that the current reverse transcriptase modifications are based on temperature-sensitive mutations and inactivation of RNase activity. The development temperature of zebrafish is generally 28 °C. Therefore, it is essential to screen reverse transcriptases to maintain a high efficiency at 28 °C. Furthermore, the fusion protein of PE with too large molecular weight limits its transmission efficiency to a certain extent. In addition, a double-blind parallel experiment at multiple sites is required to determine the feasibility of this method in zebrafish.

## 13. Epilogue

Gene editing in zebrafish has undergone development from transgene to site-directed modification. Initially, transgenic technology achieved foreign gene integration by injecting naked DNA, which was groundbreaking. However, it was inefficient and uncontrollable. Thereafter, development of the transposon system provided improved efficiency, and numerous tissue-specific fluorescence-labeled zebrafish strains were developed that significantly promoted the application of zebrafish in scientific research. In particular, the Tol2 transposable element was proven to be very efficient in integrating exogenous genes into the zebrafish genome [11]. Transposon-mediated random insertion can be used for forward genetic screening in zebrafish to identify genes that affect specific biological processes in embryos and adults. The most critical drawback of this technique is the randomness of integration, as multi-copy insertion and non-directional integration increase the complexity of the genetic background. In 2008, ZFNs successfully generated a high mutation frequency in the locus targeted by the researchers, which caused considerable excitement regarding the universal application of this technique to zebrafish genome modification [120]. In practice, the ZFN design process is laborious and time-consuming, and numerous experiments are required to screen out specific ZFNs. Moreover, the process is costly, limiting its application in zebrafish. The subsequently developed TALEN has fewer limitations, and studies have shown that compared with ZFN, the mutagenesis rate of TALEN is significantly higher [33]. In zebrafish, TALEN has been successfully used to generate alleles with gene loss-of-functions by targeting deletions and inversions of open reading frames or entire chromosomal regions. In addition to the functional deletion of genes, genome engineering using ssDNA, linearized dsDNA, or circular dsDNA as templates for homologous repair has also been implemented, enabling effective KI of sequences such as LoxP sites or fluorescent markers at protein coding gene sequences [121]. Nevertheless, because both ZFN and TALEN are protein-dependent nucleic acid recognition systems, different amino acid modules must be assembled according to the different regions of nucleic acid sequences, which is relatively difficult in terms of design and is very costly. This is in contrast to the relatively cheap CRISPR system, which can target specific genome sequences by changing only a variable sequence that is 20 nt in length, and the nuclease that acts as the gene scissors does not need to be altered. In zebrafish, the CRISPR system is highly efficient at cutting target sequences, with some targets reaching an efficiency close to 100%. The CRISPR system has achieved efficient gene knockout and site-specific KI in zebrafish since its development, significantly promoting gene-editing technology and scientific research on this vertebrate model. Nevertheless, researchers are not satisfied with the random mutations of the targeted cutting. Therefore, repair templates with nucleotide changes and homologous arms have been synthesized into single- or double-stranded donor DNA for co-injecting with Cas9 mRNA and sgRNA into zebrafish embryos to achieve site-specific gene editing. However, because of the complexity of DNA donor design, toxicity, and the low efficiency of HR-mediated KI, editing the zebrafish genome accurately and arbitrarily is a challenging task. At present, gene editing is increasingly inclined to target sequences without inducing DSBs and to adopt DNA-free components. The authors recently used the D10A Cas9n system for the first time to achieve accurate and efficient long fragment gene KI editing in zebrafish, avoiding numerous problems such as off-targets caused by DSBs and genomic instability. In addition, recently developed base editing nucleases provide an excellent method of gene editing for point mutations in zebrafish. Indeed, a set of CBEs including BE4max and AncBE4max was applied and achieved ideal efficiency in zebrafish [96]. Although the BE can produce four types of transformation mutations, there are numerous types of gene mutations that cannot be recognized by this method. More recently, the CRISPR genome project has added a new member, the PE, which can be programmed to perform precise nucleotide substitutions or deletions without the requirement of DNA donor templates and DSB production. In principle, prime editing can be used to construct almost all genetic mutation models of zebrafish associated with human diseases. Although the application prospects of this technology in zebrafish are broad, the process needs to be further optimized.

## Figures and Tables

**Table 2:** Summary of gene fixed point-oriented reconstruction studies in zebrafish.

Targeting System	Programmable Manner	Integration Mechanism	Donor Type	Insertion	Germline Transmission Rate	Disadvantage(s)	Advantage(s)	References
TALENs	DSB	HR	Linearizeds DNA	EGFP	~1.5%	Disruption of endogenous gene/Low efficiency	Large fragment insertion	[38]
CRISPR/Cas9	DSB	NHEJ	Plasmid	Gal4/RFP	~12%	Plasmid backbone insertion/unwanted indels	Large fragment insertion/easy donor design	[74]
CRISPR/Cas9	DSB	HR	Plasmid	Single base	~11%	Short-fragment insertion/unwanted indels	Correction of mismatches/target mutation	[79]
CRISPR/Cas9	DSB	NHEJ	Plasmid	EGFP	~12% (after GFP pre-screen)	Plasmid backbone insertion/unwanted indels	Large fragment insertion	[86]
CRISPR/Cas9	DSB	NHEJ	Plasmid	Venus	~20%	Unwanted indels	Large fragment insertion	[87]
CRISPR/Cas9	DSB	MMEJ	Plasmid	mCheery	~20.7%	Unwanted indels	Large fragment Insertion	[88]
TALENs	DSB	NHEJ/HR	ssDNA	LoxP	~10%	Short -fragment insertion/unwanted indels	Easy to synthesize and manipulate	[89]
CRISPR/Cas9	DSB	HR	ssDNA	Single base	~2.1%	Short-fragment insertion/unwanted indels	Correction of mismatches/point mutation	[90]
CRISPR/Cas9	DSB	HDR	ssDNA	Single base	31.8%	Unwanted indels	Correction of mismatches/point mutation	[91]
CRISPR/Cas9	DSB	HR	Plasmid	KalTA4	8%	Short-fragment insertion/unwanted indels	Large fragment insertion	[92]
CRISPR/Cas9	Nick	HR	Plasmid	GFAP	11.1%	Difficult donor design	Precise and large fragment insertion/without DSB	[93]
CRISPR/Cas9	Nick	BE system	/	C:G to T:A	7–37%	Unwanted indels/off-target risk	Without DNA template and DSB	[94]
CRISPR/Cas9	Nick	ABEmax	/	A-G	25–58%	Unwanted indels/off-target risk	Without DNA template and DSB	[95]
CRISPR/Cas9	Nick	AncBE4max	/	C:G to T:A	7.9%	Unwanted indels /off-target risk	Without DNA template and DSB	[96]
CRISPR/Cas9	Nick	PE system	PegRNA	Short-fragment insertions/deletions	30% (somatic mutations)	Short-fragment editing/unwanted indels	Without DNA template and DSB	[97]

## Data Availability

No new data were created or analyzed in this study. Data sharing is not applicable to this article.

## Abbreviation

ABE	adenine base editor
alt-NHEJ	alternative NHEJ
BE	base editor
Cas9n	modified Cas9 nickases
CBE	cytosine base editor
c-NHEJ	classical NHEJ
CRISPR	clustered regularly interspaced short palindromic repeats
CRISPR/Cas	clustered regularly interspaced short palindromic repeats and CRISPR-associated
crRNA	CRISPR RNA
dCas9	dead Cas9
DSB	double-strand break
dsDNA	double-stranded DNA
EGFP	enhanced green fluorescent protein
GCR	genetic compensation response
GFP	green fluorescent protein
gRNA	guide RNA
HMEJ	homology-mediated end joining
HR	homologous recombination
KI	knock-in
mloxP	modified loxP
MMEJ	microhomology-mediated end joining
MRN	MRE11/RAD/NBS1
NEO	nickase-based homologous recombination enhanced by RecOFAR factors
NGS	next-generation sequencing
NHEJ	non-homologous end joining
NLS	nuclear localization signal
OPEN	oligomerized pool engineering
PCR	polymerase chain reaction
PE	prime editor
pegRNA	prime editing guide RNA
RACE	rapid amplification of cDNA ends
RFP	red fluorescent protein
RGNs	RNA-guided nucleases
RNP	ribonucleoprotein
sgRNA	single guide RNA
ssDNA	single-stranded DNA
ssODN	single-stranded oligonucleotide
TALE	transcription activator-like effector
TALEN	transcription activator-like effector nuclease
TF IIIA	transcription factor IIIA
TILLING	targeting induced local lesions in genomes
ZFN	zinc finger nucleases
ZFP	zinc finger protein
zLOST	zebrafish long single-stranded DNA template
